# Separation of Heterotrophic Microalgae *Crypthecodinium cohnii* by Dielectrophoresis

**DOI:** 10.3389/fbioe.2022.855035

**Published:** 2022-05-23

**Authors:** Mario Birkholz, Danai Eleni Malti, Stephan Hartmann, Peter Neubauer

**Affiliations:** ^1^ IHP—Leibniz-Institut für Innovative Mikroelektronik, Frankfurt, Germany; ^2^ Department of Biotechnology, Chair of Bioprocess Engineering, Technische Universität Berlin, Berlin, Germany

**Keywords:** dielectrophoresis, cell separation, bioelectronics, microalgae, lipid content, microfluidics, *Crypthecodinium cohnii*, cell size distribution

## Abstract

Microalgae constitute an abundant source of poly-unsaturated fatty acids which are applied in various biotechnological fields such as pharmaceuticals and food supplement. Separating microalgae cells with respect to their lipid content would establish a relevant *at-line* analytical technique. The present study demonstrates an electrical approach for the separation of the lipid-producing microalgae *Crypthecodinium cohnii* using the effect of dielectrophoresis (DEP) in a microfluidic flow cell. Microalgae were cultivated for 8 days, while cell growth was characterized by optical density, dry cell weight, glucose concentration and lipid content *via* fluorescence microscopy. The size distribution of cells during cultivation was thoroughly investigated, since the DEP force scales with cell volume, but also depends on lipid content *via* cell electrophysiological constants. Thus, the challenge was to deconvolute one separation effect from the other, while the electrical cell constants of *C. cohnii* are not known yet. The DEP-dependent separation was realized by slanted top-bottom electrodes with the flowing cell suspension between them. Turning on the voltage deflected the cells from their initial path as determined by the streaming and thus changed their direction of flow. The separation efficiency of DEP was tested for various electrical field strengths and its performance was determined by quantitative analysis of optical and fluorescence videos. It could be shown for all size groups that the most lipid-containing cells were always subject to DEP separation and that the method is thus not only suitable for process analysis, but also for strain selection of the most productive cell lines.

## 1 Introduction

The separation of specific cells from a heterogeneous ensemble or the isolation and characterization of single cells are common tasks in biotechnology (Pethig, 2016; Li and Anand, 2018; Henslee, 2020). In recent years the application of dielectrophoresis (DEP) to these processes has been increasingly investigated, in particular, for cell separation (Markx and Pethig, 1995; Hughes, 2016). The dielectrophoretic effect operates on biological cells by inducing a dipole moment ***p***_*in*_ *via* an electrical field ***E***, causing a force that scales with ***p***_*in*_ and the inhomogeneous part of ***E*** as expressed by its gradient, resulting in ***F***_*DEP*_ = ***p***_*in*_ **∇*E***. The effect may be elicited, however, on mesoscopic length scales only and thus requires the combination with microfluidics and lab-on-chip (LOC) set-ups, into which electrical electrodes have to be integrated by microsystem technologies (Birkholz et al., 2016; Mathew et al., 2016; Ozdalgic et al., 2021). The use of DEP is therefore envisioned primarily for miniaturized LOC systems, although few commercial applications have reached the market to date (Pethig, 2016). Interesting fields of application have been explored, which however mostly address biomedical (Alazzam et al., 2011; Faraghat et al., 2017; Çağlayan et al., 2020) and less often biotechnological needs such as characterization of CHO cells (Fazelkhah et al., 2019), DNA (Viefhues and Eichhorn, 2017), bacteria (Páez-Avilés et al., 2016) or microalgae (Abt et al., 2020).

In this work, the application of dielectrophoresis to the separation of the lipid-producing microalgae *Crypthecodinium cohnii* (Marba-Ardebol et al., 2017) was investigated. The microalgae produces docosahexaenoic acid (DHA), which belongs to the group of physiologically relevant *ω*-3 fatty acids or more general to the group of poly-unsaturated fatty acids (PUFA) (Mendes et al., 2009). The bio-based production of PUFAs is being considered for feed production for aquaculture to meet future food demands of an increasing world population, especially for food fish. Here, the question was investigated to which extent *C. cohnii* cells with different PUFA content may be separated by DEP in *at-line* monitoring scenarios.

Eukaryotic *C. cohnii* belong to the family of dinoflagellates, i.e., their protist cells dispose of a transversal and a longitudinal flagellum for movements in pelagic waters enabling individual cells to reach propagation speeds of up to 145 μm s^−1^ (Fenchel, 2001; Velho Rodrigues et al., 2021). The genome of *C. cohnii* contains a large number of 95–100 chromosomes and about 7 pg DNA in its nucleus (Bhaud et al., 1991; Soyer-Gobillard and Dolan, 2015). The microorganisms feed heterotrophically and form PUFAs as a food reserve under sufficient food supply. When nutrient supply is low, lipids are consumed for energy metabolism. Lipids are not stored in dedicated reserve organelles, but form vesicles in the cytosol. The length of the cell cycle depends on nutrient supply and is finished after 12–24 h (Bhaud et al., 1991; Kwok and Wong, 2003).

**FIGURE 1:**
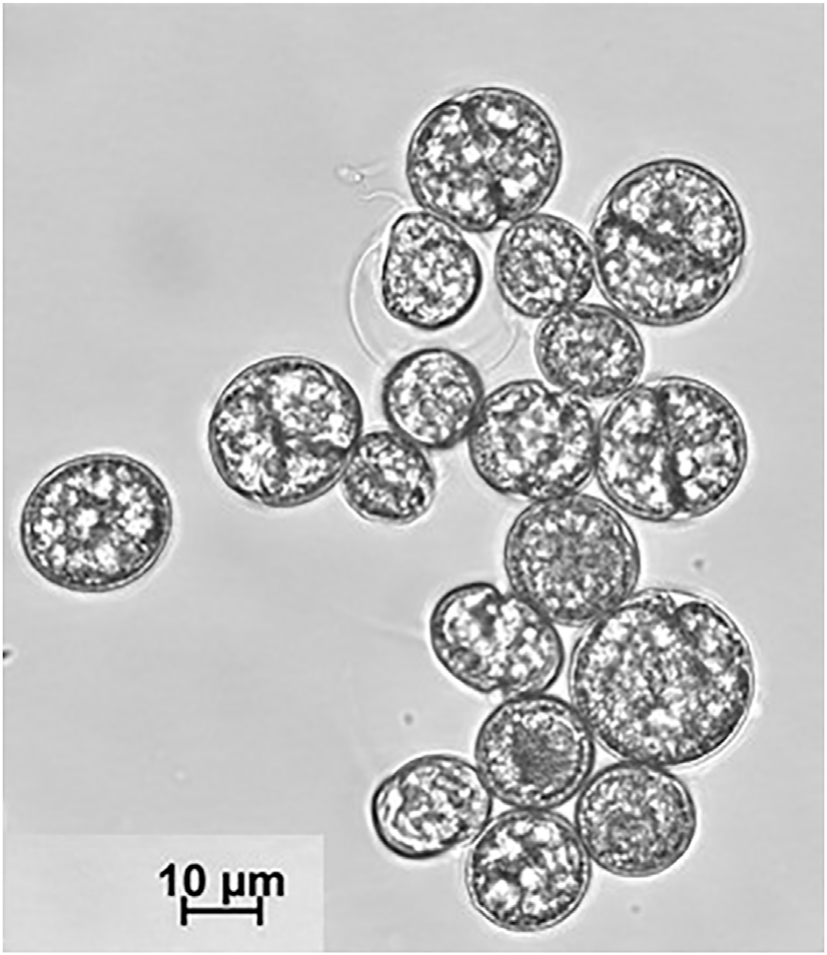
Cutout from an optical micrograph of *C. cohnii* cell suspension taken on day 4 of cultivation.

The DEP force ***F***_*DEP*_ scales with the size or volume of the polarizable particle as well as with electrical properties. Both features are important for the study performed here, since *C. cohnii* populations show significant size variations as displayed in Figure 1, which depicts a typical microscope image of a cell suspension under investigation. In addition, the dielectric constants ε and conductivities σ of algae depend significantly on lipid content (Abt et al., 2020), causing the Clausius-Mossotti factor *K*_*CM*_ = (ε*_p_−ε*_m_)/(ε*_p_ + 2ε*_m_) to vary accordingly. Here, ε* stands for the complex permittivity ε* = ε−iσ/(2π*f*), indices *p* and *m* indicate particle (or cell) and suspension medium, while the frequency *f* attains typical values in the kHz- to MHz-range in DEP experiments. The real part of ***K***_*CM*_ enters as a factor the DEP force equation and is of particular interest, since its frequency dependence determines ***F***_*DEP*_ to become either attractive or repellent, which is named positive or negative DEP.

In this study, the separability of *C. cohnii* was investigated using an electrode arrangement in the microfluidic channel that exploits the deflection of cells from their trajectories, which is otherwise imposed by the flow force field ***F***_*flow*_. For this purpose, only negative DEP was used. The focus was on the so-far uninvestigated question of whether and to what extent the separation by lipid content can be deconvoluted from the separation by size and what quantitative statements can be derived from the separated fractions. Characterization of lipid content was performed by fluorescence microscopy. The results show that DEP can be implemented as a useful characterization method for *at-line* monitoring of the lipid content of a microalgae population if the deconvolution of size and property effect has been developed before.

## 2 Materials and Methods

### 2.1 Cultivation and Characterization

*C. cohnii* populations (strain PGM-1, ATCC 30772) were cultivated in sterile 100 ml Erlenmeyer flasks in M1 cultivation medium following a protocol of Hillig (Hillig et al., 2014). 2.5 ml of preculture were diluted in 25 ml M1 medium for this purpose. The latter contains (per litre): 8 g glucose (Komplet, Völklingen, Germany), 2 g yeast extract (Ohly CPT, Hamburg, Germany), 2.05 g Na_2_SO_4_ (Merck, Darmstadt, Germany), 20 g NaCl, 5.55 g MgCl_2_×6H_2_O, 0.77 g CaCl_2_×2H_2_O, 0.35 g KCl, 0.1 g NaHCO_3_ (all from Roth, Karlsruhe, Germany). The electrical conductivity of the medium was determined by a conductivity probe (LE 703 Mettler Toledo) yielding a value of 3.5 S m^−1^ on the first day of the cultivation.

Cells were cultivated as standing cultures at room temperature (∼24°C) under darkness for 8 days. In total, four cultivations A, B, C and D were performed in order to check for reproducibility of results. Microalgae cultivation frequently suffer from bacterial contaminations, which is easily detected by optical microscopy and could be avoided in this investigation by careful preparations in a thoroughly sterilized clean bench. It is important to mention that the goal of the cultivations was not to develop an optimized protocol for lipid production, but to grow a heterogeneous population in size and lipid content.

**FIGURE 2:**
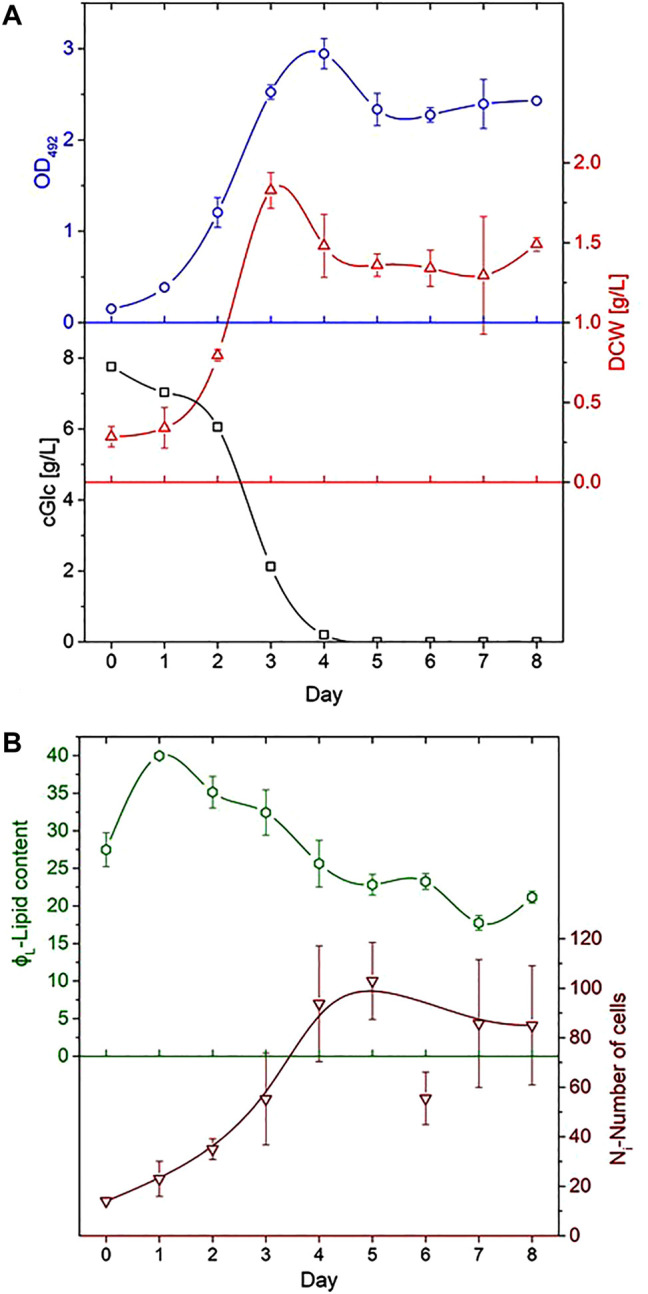
Process-analytical parameters as a function of day of cultivation. **(A)** Optical density OD as measured at 492 nm (top), dry cell weight DCW (middle) and glucose concentration CGL evaluated from the supernatant (bottom). **(B)** Plot of the average lipid content ϕ_L_ (top) and number of cells *N*_*i*_ as analysed for the determination of lipid cell size distribution (bottom). Error bars derived from the data variations in different runs; lines indicate the trend of data.

Samples were taken daily and analysed in order to estimate microalgae growth. Examination of the culture’s optical density (OD), dry cell weight (DCW) and concentration of glucose (CGL) was performed for a cultivation period of 8 days. OD was measured by a photo-spectrometer (Ultrospec 2,100 pro, Amersham Biosciences Europe, Germany) at a wavelength of 492 nm. Determination of the DCW was accomplished with the use of pre-weighed 1.5 ml dry Eppendorf tubes (MC210P-OCE, Sartorius Lab Instrument, Germany). 1 ml samples were centrifuged for 10 min at 21,500 g (Himac CT15RE, Hikoki Power 6 Tools, Germany) (the supernatant was stored at −20°C for glucose analysis), the pellet was resuspended in 0.9% NaCl and centrifuged again under the same conditions. After discarding the supernatant, the cell pellet was dried overnight and weighed in a precision balance (MC210Psat-OCE, Sartorius Lab Instruments). Glucose concentrations were derived from the analysis of the supernatant with the Cedex Bio HT, Cobas Integra 400 plus (Roche Diagnostics, Germany). Figure 2A shows the course of OD, DCW and CGL during the cultivation period with error bars derived from the standard deviations of data as obtained for the appropriate time overlaps of cultivations A-D.

To characterize cell sizes and their distributions, microscope images were acquired daily using an inverted fluorescence microscope (Eclipse Ti2-A, Nikon, Düsseldorf, Germany). Size distributions derived from the determination of geometric parameters of a large number of cells from optical micrographs by a computer-aided evaluation using an ImageJ macro (see Supplementary Material). In general, cell size distributions are presented as *x*-*y* plots with diameter classes *d*_*i*_ serving as abscissa, while the ordinate derives from the relative number *N*_*i*_/(Σ*N*_*i*_) of cells falling into this class. The usually displayed percentage is obtained by setting Σ*N*_*i*_ = 100%.

**FIGURE 3:**
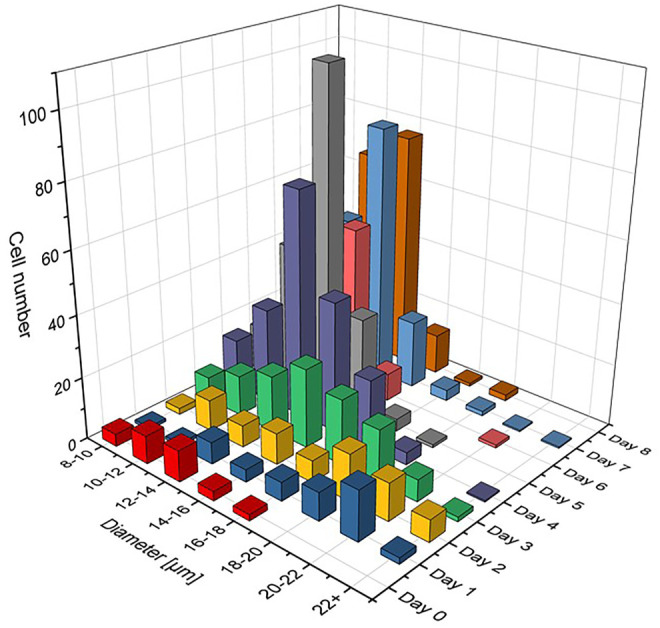
Cell size distribution functions with relative number of cells *N*_*i*_ shown for different diameter classes *d*_*i*_ having Δ*d* = 2 µm. The number of cells on each day of the cultivation is like the optical density in OD normalized graphs, Σ*N*_*i*_ = OD, so that the growth dynamics of the population from day to day can clearly be recognized.

Here, it was followed a different approach by plotting the cell count number *N*_*i*_ without normalizing to 100%. About 10 micrographs were automatically analysed for this purpose including a few hundred cells on every cultivation day. It could be shown in a separate analysis that the total number of cells was proportional to the measured value of optical density, *k*Σ*N*_*i*_ = OD. This means, that the *z* axis values shown in Figure 3 compare to a fractional OD of every size class *d*_*i*_. It can be assumed that this representation demonstrates better the growth dynamics of the algae population over time.

### 2.2 Sample Preparation

The high motility of *C. cohnii* makes microscopic investigations and microfluidic manipulation difficult. Therefore, the cells were centrifuged for 10 min at 4°C and 15,000 rpm or 21,500 g (Himac CT15RE), and right after the centrifugation the sample was washed with M1^(-)^ medium. The latter consists of the same components as M1 medium except for the yeast extract, which constitutes the nitrogen source. After this procedure, *C. cohnii* microalgae become non-motile, which may be understood from the physical loss of the longitudinal flagellum (Hu et al., 2007).

To assess the lipid content, different techniques could be applied like, for instance, FACS (Niehaus, 2019). Here, the fluorescent dye Nile red was used to stain the intracellular lipid droplets. Lipophilic Nile red emits in the 560–640 nm range in an environment of neutral or nonpolar lipids, in contrast to an emission above 650 nm corresponding to a polar lipid environment (de la Jara et al., 2003). Samples were prepared at room temperature for fluorescence microscopy by adding 10 µL of dye to a culture volume of 200 µL and investigated after an incubation time of 10 min. The microscope’s multichannel function allowed to acquire bright field and fluorescence images of the same area nearly instantaneously. For fluorescence microscopy done in this work, care was taken to ensure that all images were acquired under the same exposure conditions and that no saturation effect occurred for any pixel or cell in the detector.

**FIGURE 4:**
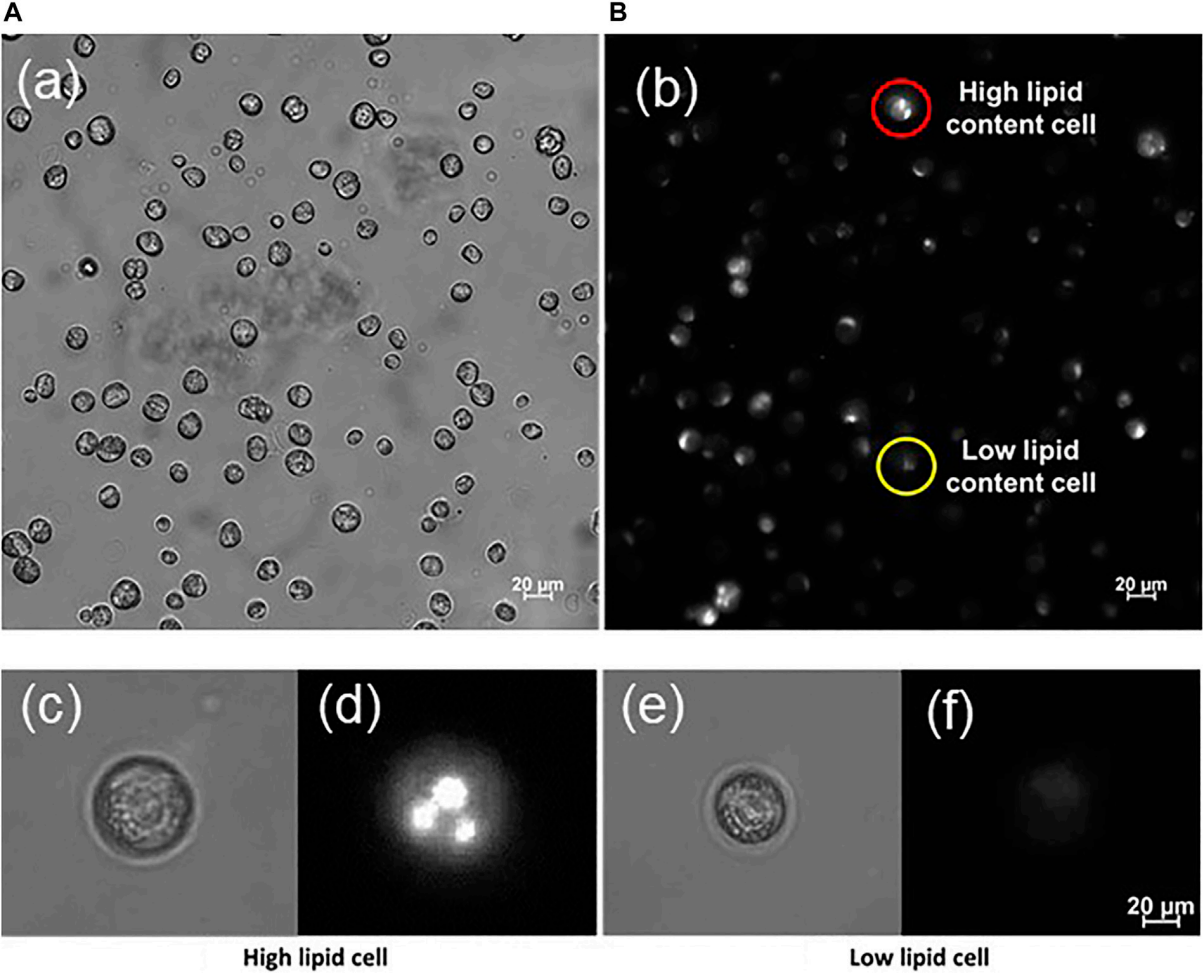
Microscopic view of immobilized *C. cohnii* cells from cultivation day number 4 in bright field **(A)** and fluorescence **(B)** mode. The cells were stained with Nile red dye to visualize their lipid content; Thus, each cell fluoresces with different intensity according to its lipid content. Cells with high lipid content appear brighter, contrary to the low lipid cells that emit less fluorescence. In both pictures **(A,B)** display the same area of the microalgae sample.—**(C–F)** Typical examples of *C. cohnii* cells showing high **(C,D)** and low lipid content **(E,F)**. The cell’s intracellular lipid seems to form droplets.

The relative lipid content of individual cells ϕ_*L*_ was determined by fluorescence microscopy in such a way that both a bright-field image and a fluorescence image were acquired (compare Figures 4A–F). From the bright-field image, the cell volume projected onto area *A* was determined as the number of pixels *p* at 600-fold magnification. In the second image, the fluorescence intensity in every pixel, 0 ≤ *I*_*F*_ ≤ 255, as measured on the same cell area was added Σ_*A*_ *I*_*F*_. The average lipid content of a cell was then determined from *ϕ*_*L*_ = (Σ_*A*_ *I*_*F*_)/*P*. Although the quantity ϕ_*L*_ describes the lipid content only on a relative scale and not in absolute units like in g L^−1^, it allows a comparison of different cells or population subgroups. Figure 2B shows the course of *N*_*i*_ and the average of ϕ_*L*_ over all cells as a function of cultivation day.

### 2.3 Microfluidic Flow Cell

**FIGURE 5:**
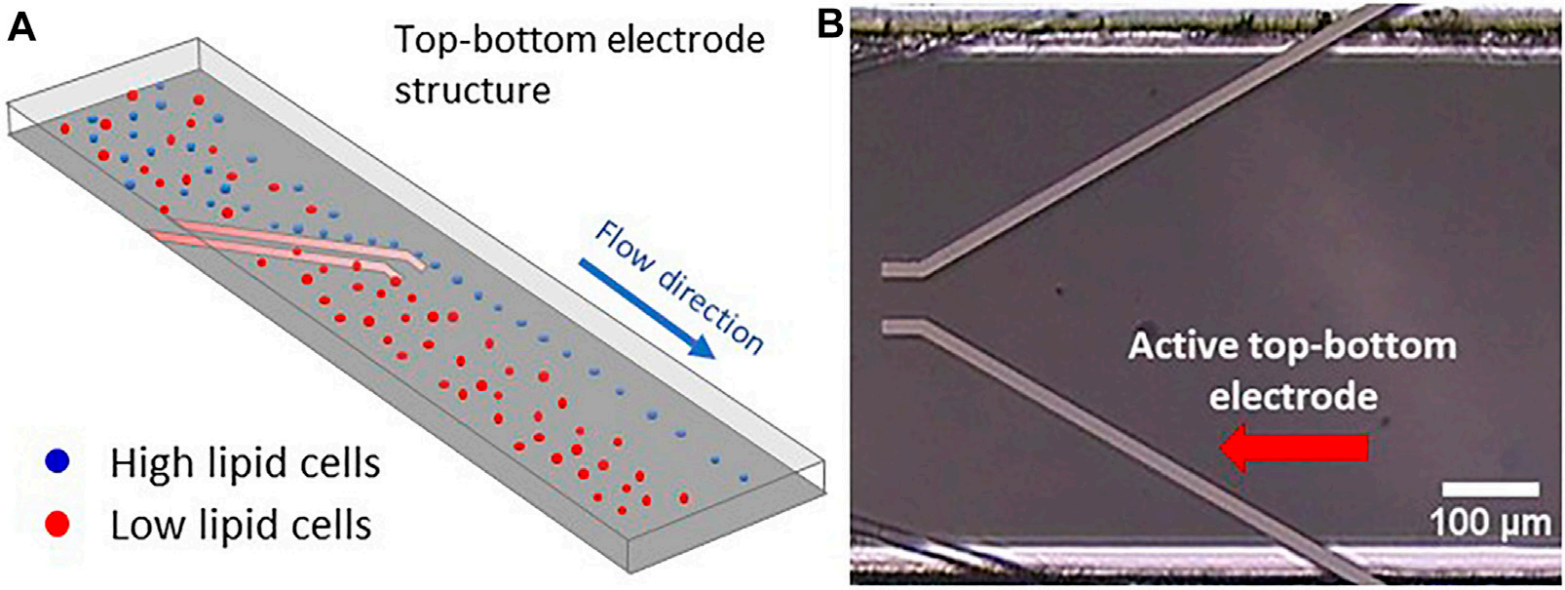
Top-bottom electrode structure with electrically conducting metal layers integrated into the top and bottom walls of the microfluidic channel. **(A)** Simulation of microalgae flow between a top-bottom electrode structure in a microfluidic flow cell. Here, the high lipid cells (blue colour) are being repelled at the electrodes edge ending up to the right outlet region while the low lipid cells are floating through the top-bottom electrode ending to the left outlet region. **(B)** Microscopic view of the microfluidic channel with two top-bottom electrode pairs. The active pair is indicated by a red arrow (in this view only the top electrodes are visible, while the bottom ones are hidden right underneath).

The basic experimental approach is shown in Figure 5A: A mixed cell population passes into the microfluidic channel toward a pair of deflector electrodes, which are thin metallic layers attached to the inner bottom and top plate of the channel. While the cells insensitive to the DEP force (red) simply flow through the region between the upper and lower electrodes, the part of the cell ensemble receptive to the DEP force (blue) is repelled by the inhomogeneous ***E*** field between electrode edges and guided to the electrodes’ end, from where on they follow the laminar flow.

The microfluidic flow cell used here consists of a single microfluidic channel of 500 µm width and 40 µm height with integrated top-bottom electrodes (GeSiM, Radeberg, Germany). These integrated electrodes are shown in vertical projection in Figure 5B, so that only the top electrodes become visible. The channel includes several electrode pairs and arrays comparable to those used by (Kirschbaum et al., 2012), and accordingly had a large number of electrical connection pins. However, for the purpose of this study, voltage was only applied to one electrode pair. The top-bottom configuration allows the cell suspension to flow between the electrodes and has already been applied to microalgae separation (Deng et al., 2014). Nevertheless, when an AC electrical signal is applied, the cells are being repelled at the electrodes’ edges, sliding next to it, since the nDEP force is acting on them. The integrated electrodes were made of a 10/120 nm thin Ti/Pt layer with a width of 20 μm, with Ti serving as adhesion layer (Birkholz et al., 2004), while Pt was exposed to the flow. Microfluidic in- and outlet of the channel were addressed by embedded ferrules with an inner diameter of 1 mm.

DEP experiments were preceded by extensive simulations using the finite element method (FEM), from which important estimates of the parameter ranges for the size of the microfluidic channel, electrode geometry, applicable electrical voltage *V*_*pp*_ (Volt peak-to-peak) and frequency *f* were determined (Gringel et al., 2018; Barai et al., 2019; Frey et al., 2021). However, unlike for other microalgae (Abt et al., 2020), the electrophysiological parameters of *C. cohnii*, i.e., electrical conductivity *σ* and dielectric constant ε of cytosol and cell membrane, are still unknown, and even more so, their functional dependence on lipid content, *σ*(*f*_*L*_) and *ε*(*f*_*L*_), has not yet been determined. *Ad hoc* assumptions had thus to be made. Before running experiments with biological cells, the commercially acquired microfluidic flow cell was tested with suspensions of polystyrene microparticles in order to check for its ability to deliver reproducible results (Boldt et al., 2021); this latter source also presents further details of the microfluidic flow cell.

### 2.4 Experimental Set-Up

**FIGURE 6:**
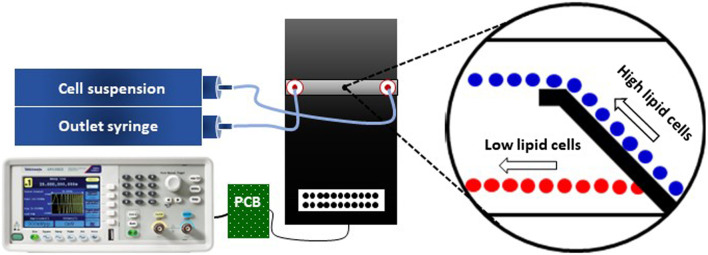
Schematic of the microfluidic setup used for *C. cohnii* separation by DEP. The setup consists of a microfluidic flow cell (black platform), two syringe pumps (inlet and outlet), a PCB and a frequency generator. The tubing between the syringe pumps and the microfluidic flow cell is symbolized by light blue-coloured lines and the cables for delivering the DEP signal are indicated by black lines. The magnified view shows the cells’ behaviour due to the DEP field, in which high lipid cells (blue colour) are being repelled at the electrodes edge while low lipid cells (red colour) are passing through the top-bottom electrode structure. For further details see (Boldt et al., 2021).

Figure 6 gives a schematic of the microfluidic set-up used for DEP separation experiments. It encompasses the microfluidic flow cell as mentioned above, two neMESYS syringe pumps (low pressure modules V2, Cetoni, Korbußen, Germany), a frequency generator (Tektronix AFG1022), and a custom-made printed circuit board (PCB) for electrically connecting the generator to the flow cell. To address the fluidic connections, a TYGON 2001 (IDEX, Germany) tubing was used with an internal diameter of 1 mm and a wall thickness of 0.84 mm, together with a PTFE tubing of 1/16” outer and 1 mm inner diameter.

In order to establish a laminar flow inside the microfluidic channel, the inlet syringe, containing the cell suspension, pumped the solution into the channel while the second syringe pulled the solution out of the channel. Both syringes were adjusted to the same flow rate of 20 μL h^−1^ and the flow was laminar and continuous during the whole experimental process. No sedimentation of cells was observed except within the syringes, where it was lifted by stirring with an enclosed magnet. QmixElements software (Cetoni, Germany) was deployed for controlling the syringe pumps.

The electrical connection between frequency generator and PCB was accomplished with a cable having a BNC connector at one end and two female pin connectors at the other. The latter were plugged to the male ones on the PCB corresponding to the targeted top-bottom electrode pair inside the microfluidic flow cell. The PCB was connected to the microfluidic chip *via* a flat ribbon cable. The DEP separation effect was investigated for an amplitude range from 1 to 12 V_pp_. The frequency *f* was set to a fixed value of 1 MHz to account for the large heterogeneity of the cell population, which varied widely in both size and lipid content. Because of the unknown electrophysiological cell constants, it was not known where the cross-over frequency *f*_*co*_ from n- to p-DEP would lie, and the cell heterogeneity would result in a wide scatter of the value anyway. Thus, preliminary FEM simulations and experiments determined that a negative DEP effect may be elicited at 1 MHz for all cells within the investigated voltage range and that good conditions for separation by size and lipid content existed in the equilibrium between drag force and DEP force. In addition, no rapture of cells was observed for these electrical parameters applied.

To monitor the cells behaviour in the DEP field the microfluidic flow cell was placed on the test holder of an inverted fluorescence microscope (Eclipse Ti2-A, Nikon) while the cell motion was captured and recorded by a high-resolution camera (DS-Qi2, Nikon) using the NIS Elements software (Nikon). Cells were illuminated through a fluorescence filter cube (Chroma DsRed ET) in the 530–560 nm range, while the detection range was set to 590–650 nm. The intensity of the fluorescence excitation lamp was always kept at 6% for static microscope images.

### 2.5 Data Analysis of Videos

Videos of the experiments were recorded to determine the separation efficiency of microalgae with the use of DEP. Cells were counted for this purpose before and after passing the top-bottom electrodes. For this purpose, the space in the channel behind the electrodes was divided into the two areas through which the cells flow after deflection and without deflection. By counting the cells in both areas and normalizing to the input number, a separation efficiency could be calculated, compare (Boldt et al., 2021).

The bright field videos were analysed with ImageJ software by creating a threshold for the cells on the obtained frames and automatically count the number of cells being repelled at the electrodes edge. Thus, the percentage of the deflected cells was calculated while results of the cells size were also obtained to deduce the size distribution of cells after being subjected to the DEP force. The analysed videos had a duration of 1 min, which allowed more than 150 cells to pass the microfluidic course captured by the microscope.

**FIGURE 7:**
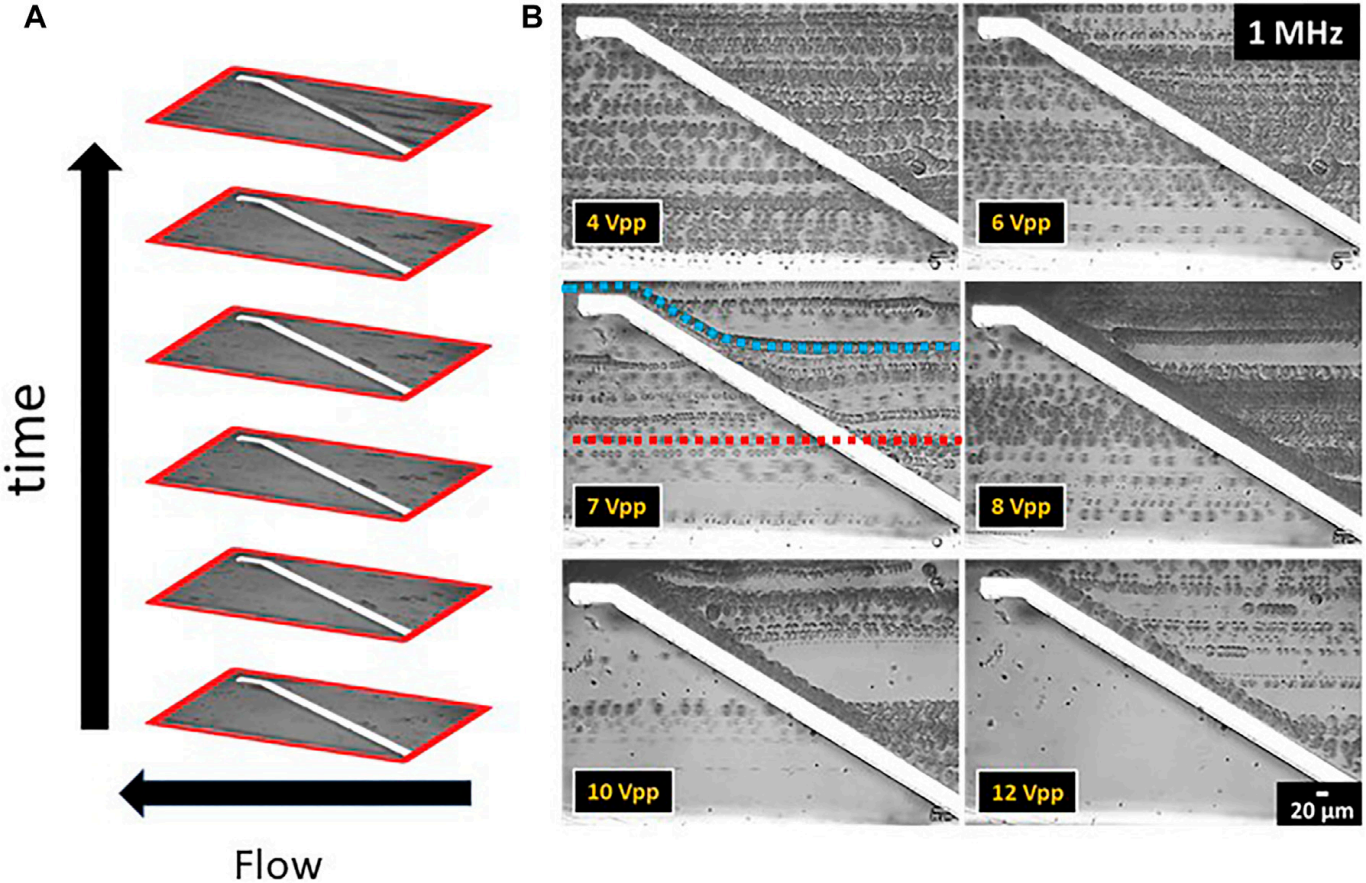
*t* stack pictures of *C. cohnii* microalgae cells in a microfluidic channel with top-bottom electrodes. The direction of flow is from right to left. **(A)** Detailed *t* stack picture format presenting the vertical placement of the photo frames over time. **(B)** *t* stack pictures of the top-bottom electrode structure showing the behaviour of *C. cohnii* cells to the DEP field for a constant frequency at 1 MHz and an amplitude range between 4–12 V_pp_. Each cell line represents the monitored flow of an individual cell as it approaches the electrode pair and flows away from it. Examples are given in the figure for 7 V_pp_, where the model trajectories for an undeflected cell (red line in the middle) and a DEP-deflected cell (blue line on top) are included.

Sections of experimental videos were converted into *t* stack pictures in order to estimate the cell separation with respect to lipid content. A *t* stack picture is a compilation of photo frames stacked one on top of each other, see Figure 7A. Videos were recorded with a rate of 35 frames per s. The *t* stack pictures shown here were composed of 50 frames each corresponding to an integration time of 1.43 s. The intensity of the fluorescence excitation lamp was kept at 20% for videos with moving cells.

## 3 Results and Discussion

### 3.1 Growth of *Crypthecodinium cohnii*

The cell populations showed comparable results with previous studies in all cultivations (Hillig et al., 2014; Marba-Ardebol et al., 2017; Niehaus, 2019). It is evident from Figure 2A that glucose was fully consumed on day 4 of the cultivation and that no major growth-supporting carbon source was available in the medium from that day on. The energy metabolism of *C. cohnii* cells thus had to switch from an external supply to the consumption of internal lipid reservoirs. Cell growth development during the cultivation is shown in Figure 3, where cell size distributions for diameter classes of Δ*d* = 2 µm (8–10, 10–12, 12–14 µm etc.) are shown for all days in the form of staggered slices. The geometric analysis of some hundred cells entered into creating this plot. *C. cohnii* cell sizes are seen to range from as small as 4 µm to rather large specimen with diameters in excess of 20 µm.

The development of distributions may be understood from the metabolic change on day 4: While the mean diameter increases from ca. 15 to ca. 24 µm on the first 2 days, smaller cells are formed by cell division in the following and the mean diameter settles at a value of ca. 18 µm. The value of the glucose concentration, which is roughly bisected from day 2–3, thus seems to be perceived by the algae cells and to slow down their expansion. From day 4, when there is no more free glucose in the medium, the lipid reserves built up until then are used as a carbon source for energy metabolism and the mean cell diameter decreases to a value of 12.5 µm. At this value, the *C. cohnii* cells probably exhibit their highest robustness towards a nutrient deficit.

### 3.2 Size Effect of the DEP Force on Microalgae

**FIGURE 8:**
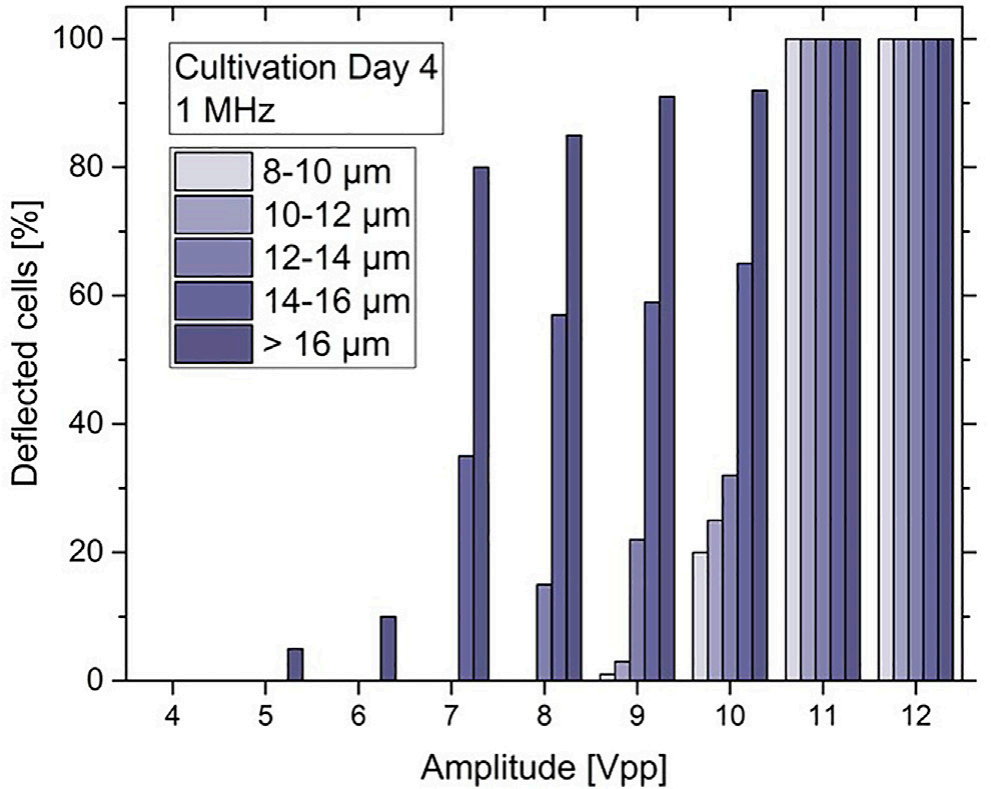
Bar chart of the deflected *C. cohnii* cells after on-set of DEP voltage exhibiting an amplitude between 4 and 12 V_pp_ and a constant frequency of 1 MHz. Cells are categorized into size groups. For large cells of more than 16 µm in diameter the nDEP effect starts to occur at 5 V_pp_. At 10 V_pp_ cells from all size groups are affected by nDEP to different amounts, while for high amplitudes at 11 and 12 V_pp_ all cells experience an nDEP force regardless of their size.

To characterize the DEP force on a heterogeneous *C. cohnii* population cells of cultivation day number 4 were taken, showing cell sizes between 8–24 µm in diameter and low to medium lipid content. For DEP experiments the above-mentioned microfluidic setup was used and a constant frequency of 1 MHz and an amplitude between 1–12 V_pp_ was applied (a typical video can be seen in the Supplementary Material Section 3). The behaviour of the cells at each amplitude range was recorded and the experimental videos were converted to *t* stack pictures (Figure 7B). Thus, it was possible to follow the trajectories of cells in the microfluidic channel and observe the DEP effect at the position of the top-bottom deflector electrode. By analysing the experimental videos, the percentage of microalgae cells that were deflected at the electrodes edge could be derived for each of the Δ*d* = 2 µm size fractions, the results of which are displayed in Figure 8.

For low amplitudes between 1–4 V_pp_ no DEP effect was detected. However, at 5 and 6 V_pp_ a slight nDEP effect appears for *C. cohnii* cells larger than 16 µm in diameter, i.e., about 15% of these cells were deflected by the DEP force. This is due to the large electrical field gradient **∇*E*** which arises at the electrode’s edge and repels the cells away from it. However, the force exerted upon them by the applied flow rate is capable to move them along its edge changing their initial direction of flow. By increasing the amplitude to 7 V_pp_ the effect of nDEP on the larger cells increases rapidly to a rate of 83% while a deflection of around 37% was observed for particles of 14–16 µm in diameter. At 10 V_pp_ cells from all size groups are being affected by the nDEP by different amounts, with larger cells recording a percentage of 93% while smaller cells, below 16 µm in diameter, are being deflected at lower rates. For high amplitudes at 11 and 12 V_pp_ total deflection for cells was observed regardless of their size.

### 3.3 Lipid Content Characterization

**FIGURE 9:**
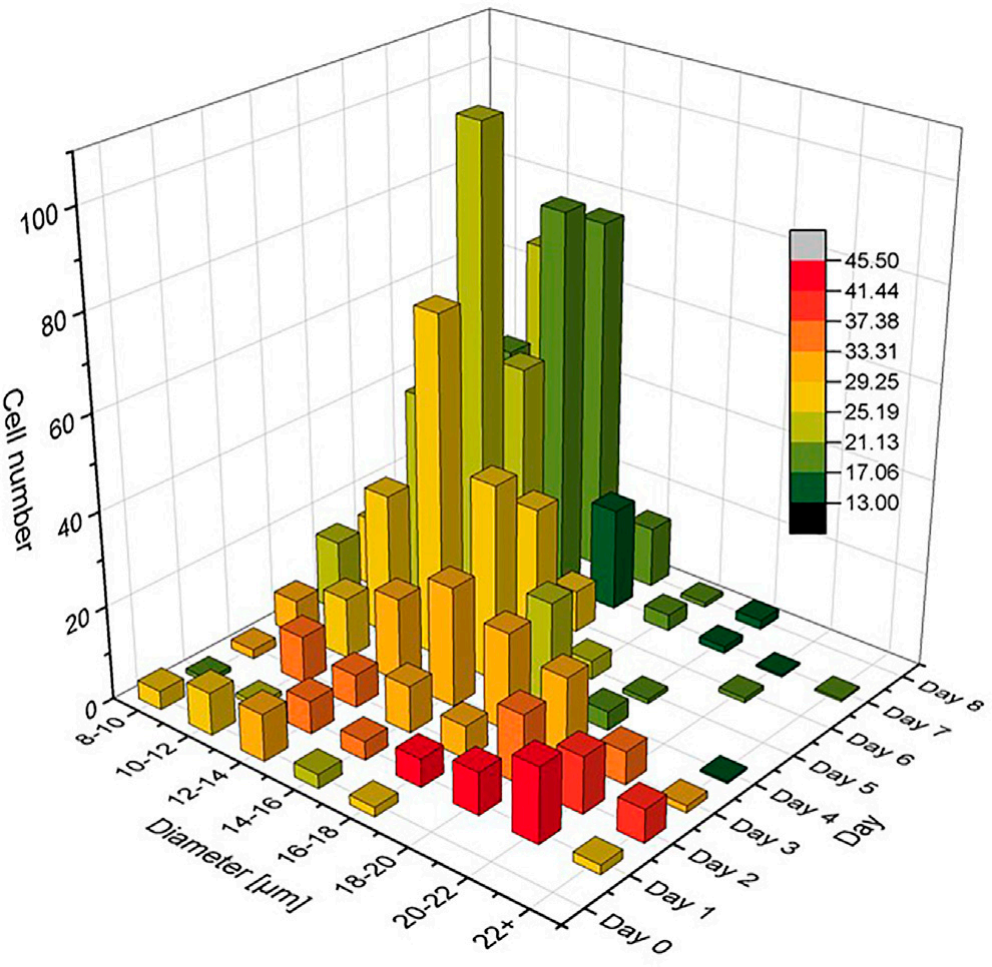
Size distribution functions as given already in Figure 3, but here in a colour code that scales with lipid content ϕ_*L*_ for each size class *d*_*i*_.

In order to understand the relationship between size and lipid content, the parameter ϕ_*L*_ was determined for all cells that had entered into Figure 3. For each bar shown in the figure, an average value for ϕ_*L*_ was determined and used as a colour code in a recoloured representation of Figure 3. As a result, Figure 9 shows the average lipid content of each cell size class as a function of cultivation day (increasing from green to red). It can be clearly seen in Figure 9 that the highest lipid contents occur on day 2 and 3, and then it decreases from day 4. The trend can be easily understood with the decrease of glucose in the culture medium, which was completely depleted from day 4. The algae cells reacted to the reduced nutrient supply by consuming the previously formed fat reserves. In this way, it was possible to generate an extremely heterogeneous population of *C. cohnii* cells, which not only differed significantly in size on day 4, for example, but also with respect to the ϕ_*L*_ parameter. Interestingly, the plot shows decreasing cell sizes after day 4, which again can be explained by the decreasing nutrient availability.

### 3.4 DEP-dependent Separation by Lipid Content

To examine the separation of *C. cohnii* microalgae with respect to lipid content cells of cultivation day number 4 with ϕ_*L*_ values up to 117 were examined in detail. The microfluidic set-up was placed under the fluorescence microscope and the cells were stained with Nile red dye so that their lipid droplets became visible in fluorescence microscopy. The experiments were performed for a constant frequency of 1 MHz and an amplitude of 10 V_pp_. The selection of these two values derived from the fact that at these particular values, deflections in all different size groups were observed.

**FIGURE 10:**
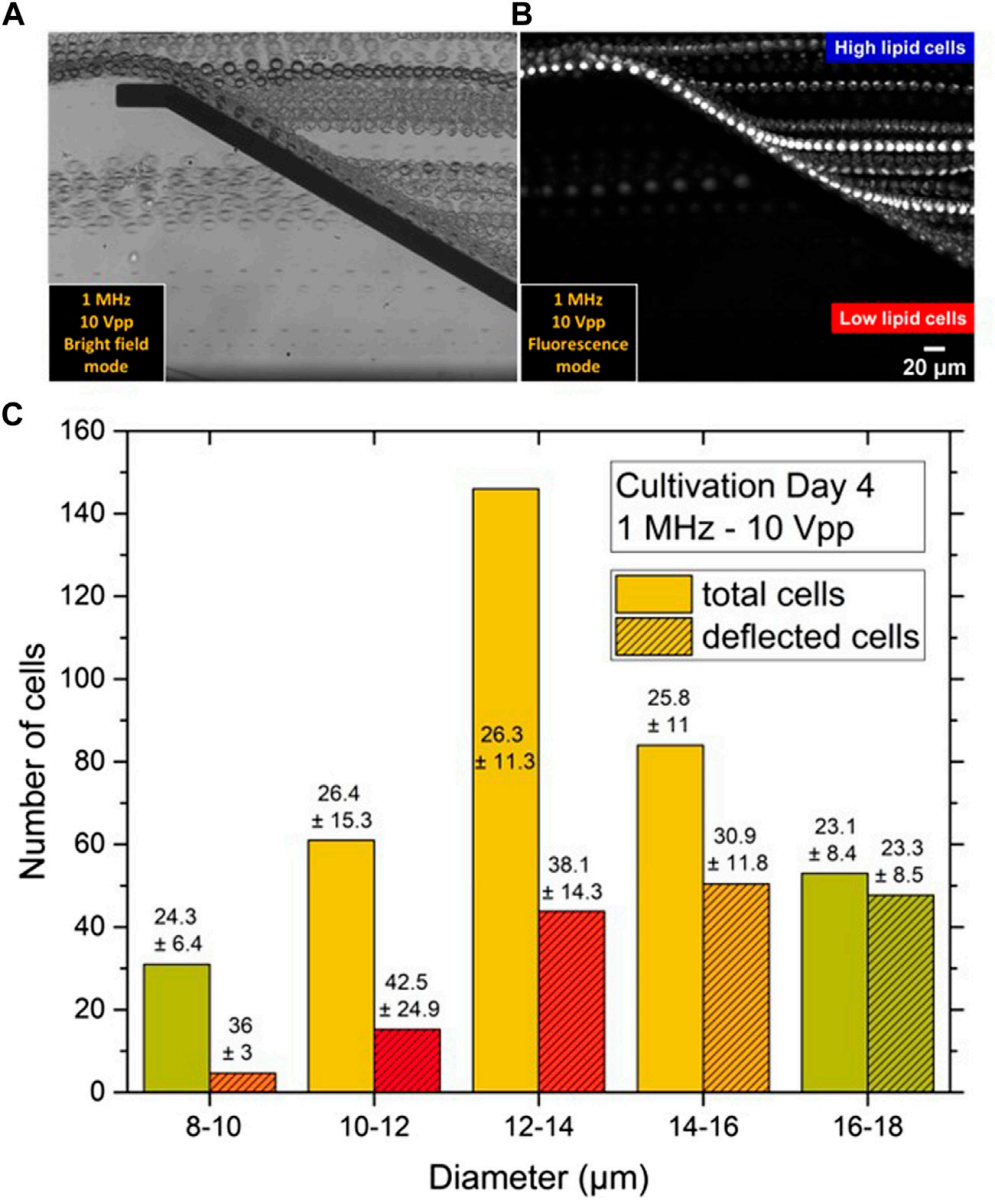
**(A)** *t* stack images of bright field and **(B)** fluorescence micrographs showing the nDEP effect for *C. cohnii* microalgae at 1 MHz and 10 V_pp_. The direction of flow is from right to left. Cells of high lipid content as affected by nDEP are repelled by the top-bottom electrode structure, separating them from low lipid cells, which are flowing between the electrode pair without altering their direction of flow. **(C)** Size distribution of cell suspension at day 4 of cultivation before and after DEP separation. Numbers stand for the mean lipid content ϕ_*L*_ of each size class and its dispersion δϕ_*L*_ *.* Unstriped bars show the initial number of cells *N*_*i*_ before DEP separation, which compares to Day 4 slices in Figures 3, 9. Striped bars stand for the number of cells as observed after DEP separation in the deflected flow. Inscribed data now indicate average ϕ_*L*_ values as determined from the high-lipid fraction of each size class, the percentage of which was derived from 10 V_pp_ values in Figure 8. The lipid content ϕ_*L*_ of deflected cells is seen to surmount that of undeflected ones for all size classes, except for the last one.

For a continuous flow of 20 μL h^−1^ high lipid content microalgae could be separated from low lipid ones, see Figure 10. Microalgae with a higher lipid content, which appear brighter under the fluorescence light, were affected by the nDEP force and repelled at the edges when they approach the top-bottom electrode structure. This is due to the large electrical field gradient developing at electrode edges, causing the repulsion of cells. However, the force exerted on the cells by the flow field is still sufficiently large to transport the cells along the electrodes causing them to change their flow direction. Microalgae with low lipid content do not appear to be subjected to any DEP forces; they maintain their initial flow direction and pass unaffected between the top-bottom electrode structures. Therefore, two different flow streams occur inside the microfluidic channel right after the electrode area: one containing the high lipid microalgae on the top side of the channel, and a second containing the low lipid microalgae on the bottom side. Both streams continue to flow in an unmixed and laminar fashion along the microfluidic channel until they reach the outlet.

The quantitative analysis of this effect was not straightforward, since it was not possible to record simultaneous videos in both brightfield and fluorescence mode. Typical video sequences recorded in each of both modes are appended as Supplementary Material. Nevertheless, to proceed beyond a purely qualitative description, the results from Figure 8 were combined with those from Figure 9. Figure 8 shows for each fraction with mean size of 9, 11, 13, 15 and 17 µm the fraction deflected by the DEP force ν at 10 V, which was 17, 22, 30, 65 and 90%, respectively. Based on the studies already done on other lipid-forming microalgae (Abt et al., 2020), it can be assumed that the deflected cells were not deflected solely because of their size but also because of their high lipid content. Indeed, the cells of each size class always showed some dispersion of lipid content δϕ_*L*_ around the mean value ϕ_*L*_. For the analysis, all cells of each size class that showed the highest ϕ_*Lmax*_ values were now selected - and so many that their number *N*_*d*_ϕ_*Lmax*_ normalized to the total number of class *N*_*d*_ corresponded exactly to the deflected fraction, n = *N*_*d*_ϕ_*Lmax*_/*N*_*d*_, as shown in Figure 8.

Figure 10C shows the size distribution of the *C. cohnii* cells on day 4 of cultivation as already displayed as one layer of Figure 3. The height of the bars again corresponds to the measured cell number *N*_*d*_ in the respective fraction. In addition, smaller bars are also given for each *d* value, which are shaded. Their height corresponds to the DEP-selected fraction ν⋅*N*_*d*_, resulting in a slightly different representation than in Figure 8, where only the ν values are plotted. Inscribed into the bars of the shaded histogram are the lipid contents ϕ_*Lmax*_ that the fraction ν of each size class exhibited.

It can be seen that the ϕ_*Lmax*_ values for the last four size classes decrease continuously from 42.5 to 23.5 (the size class 9 µm was not included in the analysis because the shaded area represents only a statistically unrepresentative number of cells). At the same time, the difference, ϕ_*Lmax*_ - ϕ_*L*_, between the lipid content of all cells in the group ϕ_*L*_ and the group with the high contents ϕ_*Lmax*_ decreases. This means that as the size increases, the lipid content can become smaller and smaller to produce a deflection effect for the cell at the DEP electrodes. While the large cells are all deflected, selection by lipid content occurs for the small cells. The results also show that the selection limits - by lipid content or size - can be set by adjusting the voltage *V*. Another control option may be the frequency *f*, but this question was not investigated in this work.

## 4 Conclusion

DEP force allows for two separation effect: by size and by electrical properties, which translates in different lipid contents in the case considered here. Both separation processes were demonstrated for the model configuration of a single channel MF LOC that made use of a top-bottom configuration of deflector electrodes integrated into the MF channel. An AC voltage supply was used exhibiting a voltage of 10 Vpp and frequency of 1 MHz, for the latter of which always a repulsion of cells from the electrodes was observed (negative DEP).

Thus, cell separation by DEP has proven to be an interesting analytical method for the characterization of lipid-producing bioprocesses. One may hardly recognize an application of the technique in downstream processes such as harvesting or extraction of particularly lipid-containing cells, because the volume turnovers in microfluidic channels are simply too low. However, the population of bioproducers, which develops very heterogeneously in a process, can be characterized by DEP as with a fingerprint. It should be possible to derive suitable analytical platforms from the LoC developed by Deng et al., which uses staggered electrodes in the microfluidic channel (Deng et al., 2014).

What would also be needed are automatic cell counters with which the number of deflected cells could be quantified, simple and effective electrical systems have already been developed for this purpose, cf. e.g., (Sobahi and Han, 2020). The combination of deflection and cell counting could then be used to develop effective protocols for controlling the DEP electrodes, which could be used to draw meaningful conclusions about the status of the lipid-producing bioprocess and which could be used for reactor control and optimization.

A further field of application also opens up for strain selection. The experiments shown indicate that cells with particularly high lipid contents can always be separated from the total population using DEP separation. In this respect, the label-free and non-invasive nature of DEP is particularly relevant, because the selected cells are still viable after separation, which distinguishes DEP from other methods such as FACS or MACS. In principle, the DEP technique is of interest whenever small, valuable cell quantities are to be handled. Thus, it is expected that the method presented here for the separation of lipid-rich microalgae—as well as other heterogeneous cell populations—will find widespread application in the future of bioprocess engineering.

## Data Availability

The raw data supporting the conclusion of this article will be made available by the authors, without undue reservation.

## Author Disclaimer

We point out the ethical conflict that this work aims at biotechnological food production, which is often motivated by the expected increase of world population. However, as outlined by the Scientists’ 2nd Warning Initiative (Ripple et al., 2017) in numerous articles, human colonization of planet Earth is increasingly accompanied by overuse of natural resources, e.g., (Finlayson et al., 2019; Cardoso et al., 2020; Washington et al., 2020; Albert et al., 2021). Therefore, this work should not be understood as suggesting that population increase can simply be managed by technical means. Rather, we think that, in addition to research programs to develop biotechnological processes for food production, there is an urgent need to initiate political measures aimed at limiting global population growth in order to secure a sustainable future for human life on Earth.
