# Supplementary material for: Separation of Heterotrophic Microalgae Crypthecodinium cohnii by Dielectrophoresis
Source: Front Bioeng Biotechnol. 2022 May 23;10:855035. doi: 10.3389/fbioe.2022.855035 (PMC9169251; doi:10.3389/fbioe.2022.855035)
Supplement: Supplementary file 1 [file DataSheet1.pdf]

Supplementary Material

1. ImageJ macro for the analysis of cell size parameters from optical microscope images

```
//manual opening of a brightfield image
//adjust the scale for the microscope images
run("Set Scale...", "distance=401 known=50 unit=µm global");
//adjust the measurements
run("Set Measurements...", "area redirect=None decimal=3");
//reduce the background to reduce disturbances outside the focal plane
run("Subtract Background...", "rolling=50 light disable");
//automatic marking of cells
setAutoThreshold("Default no-reset");
//run("Threshold...");
setAutoThreshold("Default no-reset");
//setThreshold(0, 196);
setOption("BlackBackground", false);
run("Convert to Mask");
//fill holes in the marked cells
run("Fill Holes");
//Use "watershed" plugin to separate connected cells into different objects
run("Watershed");
//Manual control of marked cells with paintbrush tool (remove moving or blurred cells, fill unmarked cell areas at cell edges and remove the microscope generated scale, if it makes problems with a cell marking)
run("Analyze Particles...", "size=50-Infinity circularity=0.60-1.00 show=Nothing display exclude")
String.copyResults();
```

2. Semiautomatic ImageJ macro for analyzing the lipid content of cells from a fluorescence and the corresponding brightfield microscope images

Part 1:

```
//manual opening of a fluorescence image in the background and the corresponding brightfield image in the foreground
//adjust the scale for all microscope images
run("Set Scale...", "distance=401 known=50 unit=µm global");
//adjust the measurements
run("Set Measurements...", "area mean redirect=None decimal=3");
//reduce the background to reduce disturbances outside the focal plane
run("Subtract Background...", "rolling=50 light disable");
//automatic marking of cells
setAutoThreshold("Default no-reset");
//run("Threshold...");
```

```
setAutoThreshold("Default no-reset");
//setThreshold(0, 196);
setOption("BlackBackground", false);
run("Convert to Mask");
//fill holes in the marked cells
run("Fill Holes");
//Use "watershed" plugin to separate connected cells into different objects
run("Watershed");
```

//Manual control of marked cells with paintbrush tool (remove moving or blurred cells, fill unmarked cell areas at cell edges and remove the microscope generated scale, if it makes problems with a cell marking)

Part:2

```
//Stack the image of the black marked cells with the fluorescence image, to create a new image in which each pixel has the higher gray value of the two images.
run("Images to Stack", "name=Stack title=[] use");
run("Z Project...", "projection=[Max Intensity]");
//threshold the stack image by marking all pixels except the gray value 255 (white background)
run("Manual Threshold...", "min=0 max=254");
//Analize the marked area
run("Analyze Particles...", "size=50-Infinity circularity=0.60-1.00 show=Nothing display exclude")
String.copyResults();
```

2.1. Supplementary videos

Video 1: Brightfield-separation
Video 2: Fluorescence-separation

Sections from a brightfield and a fluorescence microscope video showing the DEP separation effect of the active top and bottom electrode in the microfluidic channel with *C.Cohnii* cells at $10V_{pp}$ and 10 MHz.
